# Tollip interaction with STAT3: a novel mechanism to regulate human airway epithelial responses to type 2 cytokines

**DOI:** 10.1186/s12931-022-01941-x

**Published:** 2022-02-16

**Authors:** Niccolette Schaunaman, Kris Genelyn Dimasuay, Monica Kraft, Hong Wei Chu

**Affiliations:** 1 National Jewish Health, Denver, CO USA; 2 University of Arizona, Tucson, AZ USA; 3 Department of Medicine, National Jewish Health, 1400 Jackson Street, Room A639, Denver, CO 80206 USA; 4 Department of Medicine, College of Medicine, Tucson, 1501 Campbell Avenue, Office 6334, Tucson, AZ 85724 USA

**Keywords:** Tollip, STAT3, Airway epithelial cells, IL-13, Type 2 inflammation, Protein–protein interaction

## Abstract

**Background:**

Toll-interacting protein (Tollip) is one of the key negative regulators in host innate immunity. Genetic variation of Tollip has been associated with less Tollip expression and poor lung function in asthmatic patients, but little is known about the role of Tollip in human airway type 2 inflammatory response, a prominent feature in allergic asthma.

**Objective:**

Our goal was to determine the role and underlying mechanisms of Tollip in human airway epithelial responses such as eotaxin to type 2 cytokine IL-13.

**Methods:**

Tollip deficient primary human airway epithelial cells from 4 healthy donors were generated by the gene knockdown approach and stimulated with IL-13 to measure activation of transcription factor STAT3, and eotaxin-3, an eosinophilic chemokine.

**Results:**

Following IL-13 treatment, Tollip deficient cells had significantly higher levels of STAT3 activation and eotaxin-3 than the scrambled control counterpart, which was reduced by a STAT3 inhibitor. Interaction between Tollip and STAT3 proteins was identified by co-immunoprecipitation.

**Conclusion:**

Our results, for the first time, suggest that Tollip inhibits excessive eotaxin-3 induction by IL-13, in part through the interaction and inhibition of STAT3. These findings lend evidence to the potential of a STAT3 inhibitor as a therapeutic target, especially for type 2 inflammation-high asthmatics with Tollip deficiency.

## Background

Toll-interacting protein (Tollip) is a multifunctional immune regulator under both physiological and pathological conditions [1]. One of the major functions of Tollip in innate immunity is to prevent excessive pro-inflammatory responses [2]. Reduced Tollip expression and subsequent dysfunction of the biological system due to genetic variation or pathological conditions have been reported in various human diseases including cancer, Alzheimer’s disease, tuberculosis, atopic dermatitis, pulmonary fibrosis and infections [3–8]. Our recent work demonstrated that Tollip single nucleotide polymorphism (SNP) rs5743899 is associated with reduced Tollip expression in airway epithelial cells and poor lung function in asthma patients [9]. More specifically, asthma subjects carrying the G allele (AA or AG) of rs5743899 had significantly lower FEV1/FVC ratio than those carrying the A allele (AA), indicating airflow limitation. Mechanistically, human airway epithelial cells with AG or GG of rs5743899 expressed less Tollip, but produced significantly higher levels of neutrophilic chemokine IL-8 and eosinophilic chemokine eotaxin-3 than the cells with AA following the stimulation with IL-13 and rhinovirus, a major risk factor in asthma exacerbations. Asthma is a heterogeneous chronic respiratory condition that encompasses several different endotypes [10]. One such endotype is type 2-high asthma, that is distinguished by the presence of type 2 cytokines (e.g. IL-13), and accumulation and activation of eosinophils [11–13], and increased severity of the disease [12, 14]. Regulation of eosinophilic inflammation by innate immune factors has not been well studied. The major goal of this report is to determine a novel role of Tollip in the production of pro-eosinophilic mediator eotaxin-3 in human primary airway epithelial cells, a first line host defense mechanism in respiratory diseases. IL-13 is known to induce eosinophilic inflammation (e.g. production of eotaxin-3) by activating transcription factor STAT6 in airway epithelial cells [15]. Recently, STAT3, another member of the STAT transcription factor family [16], has been found to be activated by IL-13 by our group and others [17, 18]. Moreover, airway epithelial STAT3 has been shown to be essential in initiating type 2 inflammation in an allergic asthma mouse model [19]. Whether and how Tollip may regulate airway epithelial responses (e.g. eotaxin production) to IL-13 remain unclear. In this report, we presented a novel hypothesis that Tollip interacts with STAT3 to inhibit its activation and the ensuing type 2 inflammation (e.g. eotaxin production).

## Methods

### Isolation of human tracheobronchial epithelial (HTBE) cells

HTBE cells were isolated as previously described [20] from de-identified donor lungs that were not suitable for transplantation. The four selected donors were nonsmokers with no prior history of lung disease (Table 1). The Institutional Research Board at National Jewish Health approved this research.

**Table 1:** Subject demographics

Subject	Gender	Age (years)	Smoking history	Cause of death
1	Male	36	Never	Anoxia
2	Female	68	Never	Stroke
3	Male	50	Non-smoker (> 12 years)	Stroke
4	Male	60	Never	Stroke

### Lentivirus-mediated knockdown of Tollip in HTBE cells

HTBE cells from four healthy donors were transduced with human Tollip or control (scrambled) short hairpin RNA (shRNA) obtained from Genecopoeia (Bethesda, MD) as we have previously described [17]. Briefly, 293FT cells grown in a 60 mm culture dish in Dulbecco’s modified Eagles Medium containing 10% FBS, with no antibiotics, were transduced with the lentivirus constructs to produce supernatants containing packaged lentivirus. Lentivirus-containing supernatants were then used to transduce HTBE cells in six-well plates [17, 20, 21] to generate Tollip deficient and Tollip-sufficient cells, which were selected and further expanded on puromycin resistant irradiated 3T3 fibroblasts in the presence of 1 µg/ml puromycin and a Rho kinase inhibitor.

### Treatment of HTBE cells

HTBE cells from four healthy donors, either transduced or not-transduced, were seeded onto collagen coated 24-well plates at a seeding density of 5 × 10^4^ cells/well in 500 µl of BronchiaLife (Lifeline Cell Technologies, Frederick, MD) and grown in submerged culture until 80–90% confluency. For experiments with IL-13 alone, cells were treated with 10 ng/ml of IL-13 (PeproTech, Rocky Hill, NJ) or 0.01% BSA as a control in BronchiaLife. For STAT3 inhibitor experiments, cells were pre-treated with 50 µM of STAT3 Inhibitor VI (S31-201, MilliporeSigma, Burlington, MA) for 30 min or 0.5% DMSO in BronchiaLife, after which, 10 ng/ml of IL-13 was added to appropriate wells. We chose the IL-13 dose and duration of treatment based on our previous publication [18]. Cells and supernatants from both experiments were harvested 24 and 48 h after IL-13 treatment.

### ELISA

Eotaxin-3 was measured in supernatants using a Human CCL26/Eotaxin-3 DuoSet ELISA kit (R&D Systems, Minneapolis, MN).

### Western blot analysis

Cells were lysed in radioimmunoprecipitation assay (RIPA) buffer containing protease and phosphatase inhibitors (ThermoFisher, Waltham, MA). Equal amounts of total protein from each cell culture condition were separated by SDS-PAGE, transferred onto PVDF membranes, blocked with blocking buffer, and incubated with the following primary antibodies overnight at 4 °C: Tollip monoclonal antibody (Enzo Life Sciences International, Farmingdale, NY), Phospho-Stat3 antibody (Tyr705, Cell Signaling Technology, Danvers, MA), Stat3 antibody (Cell Signaling Technology), and Beta-Actin antibody (Santa Cruz Biotechnology, Dallas, TX). After washes in PBS with 0.1% Tween-20, membranes were incubated with the appropriate horseradish peroxidase (HRP)-linked secondary antibodies and developed using a Fotodyne imaging system (Fotodyne, Inc., Harland WI).

Densitometry was performed using the NIH ImageJ software.

### Statistical analysis

Student’s *t* test was used for two group comparisons. A P-value of < 0.05 was considered significant.

### Tollip deficiency promotes STAT3 activation and type 2 inflammatory responses

**Fig. 1:**
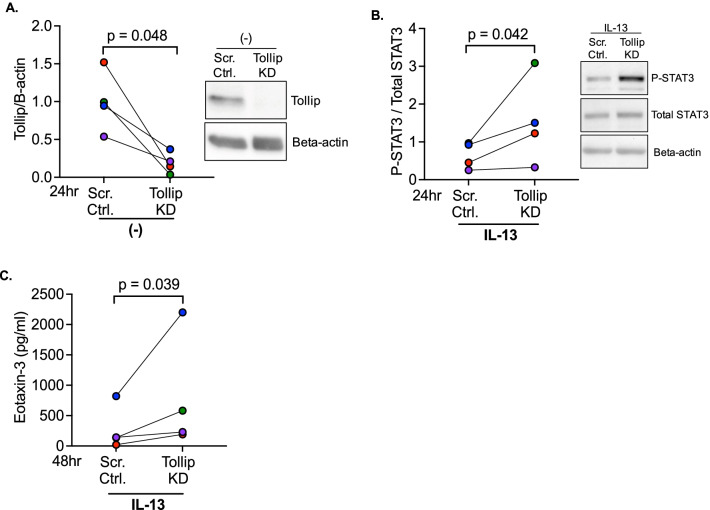
Tollip deficiency promotes STAT3 activation and inflammatory response to IL-13. **A** Western blot image showing Tollip knockdown (KD) using the shRNA approach in tracheobronchial epithelial cells. Data was normalized to the scrambled control. **B** Increased STAT3 activation in Tollip KD cells vs. scrambled control after 24 h of IL-13 treatment. Data was normalized to the scrambled control cells with IL-13 treatment. **C** Significantly higher eotaxin-3 levels in Tollip KD cells vs. scrambled control cells after 48 h of IL-13 treatment. P-values represent a paired student’s *t*-test. Data are from four healthy human donors. Each dot represents one donor. Data from one donor is the mean of three technical replicates

We utilized Tollip deficient (knockdown, KD) primary human HTBE cells (Fig. 1A) from four healthy donors to explicitly demonstrate a role of Tollip in airway epithelial type 2 inflammatory response. As shown in Fig. 1B, Tollip KD cells have significantly higher levels of STAT3 activation (phosphorylation) than the scrambled control cells after 24 h of IL-13 treatment. Subsequently (48 h after IL-13 treatment), Tollip KD cells produced more eotaxin-3, a potent eosinophil chemokine, than the control (scrambled shRNA) cells (Fig. 1C). Of note, eotaxin-3 was not detectable in cells without IL-13 treatment. Our data provided the first evidence that Tollip deficiency exaggerated type 2 cytokine response, including excessive STAT3 activation and production of eotaxin-3.

### STAT3 activation is responsible for exaggerated type 2 inflammatory response in Tollip deficient cells

**Fig. 2:**
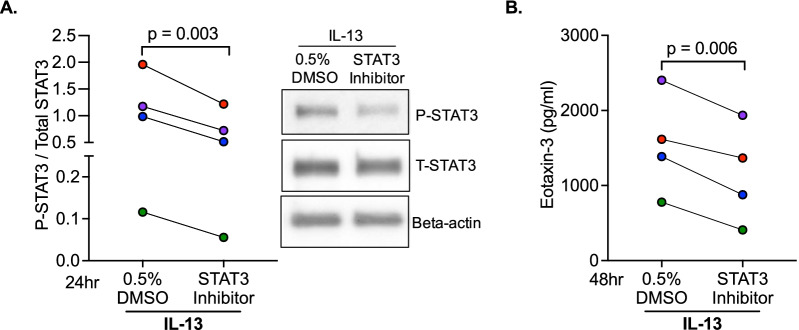
STAT3 inhibition reduces the type 2 inflammatory response in IL-13 treated Tollip sufficient tracheobronchial epithelial cells. **A** IL-13 treatment increased activation/phosphorylation of STAT3, which was inhibited by a STAT3 inhibitor. Data was normalized to the IL-13 treatment with 0.5% DMSO. **B** A STAT3 inhibitor significantly reduced the levels of IL-13-induced eotaxin-3 in supernatants. P-values represent a paired student’s *t*-test. Data are from four healthy human donors. Each dot represents one donor. Data from one donor is the mean of three technical replicates

To determine if STAT3 activation is responsible for eotaxin-3 production, we first inhibited STAT3 activity using a highly selective inhibitor (S31-201) in Tollip sufficient cells. As shown in Fig. 2A, the STAT3 inhibitor significantly reduced STAT3 activity 24 h after IL-13 treatment, which was followed by significant reduction of eotaxin-3 protein at 48 h of IL-13 treatment (Fig. 2B).

**Fig. 3:**
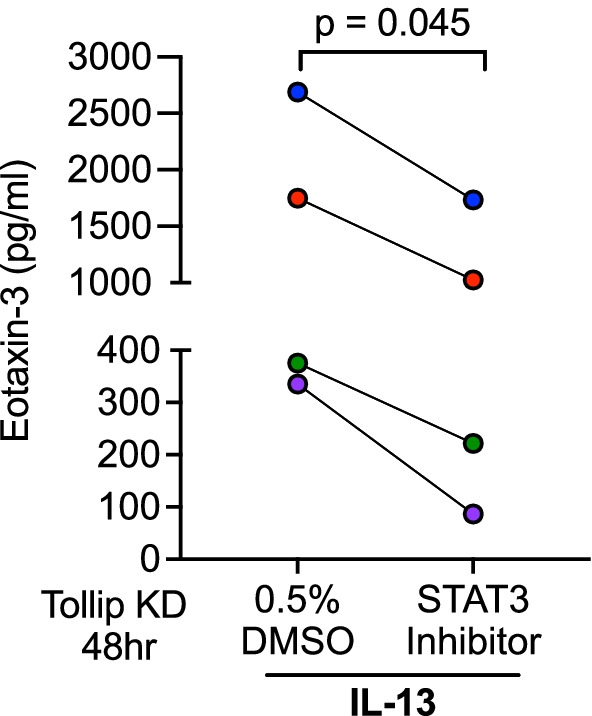
STAT3 inhibition reduces the excessive type 2 inflammatory response in Tollip deficient human tracheobronchial cells. STAT3 inhibition significantly reduced eotaxin-3 levels in supernatants of Tollip knockdown cells after 48 h of IL-13 treatment. P-values represent a paired student’s *t*-test. Data are from four healthy human donors. Each dot represents one donor. Data from one donor is the mean of three technical replicates

Next, we determined if more eotaxin-3 production in IL-13-treated Tollip KD cells is dependent on excessive STAT3 activation. As shown in Fig. 3, IL-13 mediated excessive eotaxin-3 production in Tollip KD cells were significantly reduced by a STAT3 inhibitor.

While our results continue to provide more evidence of the involvement of STAT3 in type 2 inflammation, for the first time we showed that Tollip is able to reduce IL-13-induced type 2 inflammatory response (i.e. eotaxin-3) through the inhibition of STAT3 activation. By using a conditional STAT3 knockout mouse model, Simeone-Penney and colleagues demonstrated that STAT3 signaling in airway epithelium was required for an eosinophilic inflammatory response following allergen challenges [19]. Others have shown that STAT3 is required for T helper 2 cell development [22]. Together, our data and others support the potential of using STAT3 inhibitors as a potential therapy in combating excessive type 2 inflammatory response seen in patients with Tollip deficiency.

### Tollip interacts with STAT3

**Fig. 4:**
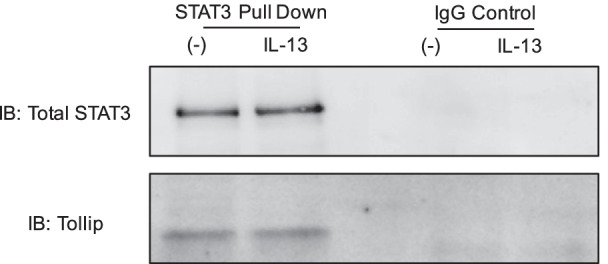
Interaction of Tollip and STAT3. Co-immunoprecipitation of STAT3 and Tollip after 24 h of IL-13 treatment in cultured normal human tracheobronchial epithelial cells. *IB* immunoblotting

To determine how Tollip may inhibit STAT3 activation, we sought to identify if Tollip interacts with STAT3 at the protein level. As shown in Fig. 4, STAT3 was successfully pulled down via co-immunoprecipitation (Co-IP) assay. Importantly, the STAT3 Co-IP protein complex contained Tollip, indicating a protein–protein interaction of Tollip and STAT3. Thus, we are the first to identify a novel protein–protein interaction of STAT3 and Tollip. Tollip is comprised of three domains. At present, we do not know how Tollip interacts with or binds to STAT3. To clearly demonstrate how Tollip regulates IL-13-medaited STAT3 activation and function, future studies will be needed to clarify which Tollip protein domain is responsible for its interaction with STAT3, and if Tollip/STAT3 interaction subsequently promotes the degradation of STAT3.

As data from our current study are from healthy donors that have not been genotyped for Tollip SNP rs5743899, future studies can include airway epithelial cells from human subjects with different Tollip genotypes to study whether varying levels of Tollip expression are associated with STAT3 activation levels and pro-inflammatory responses following type 2 cytokine stimulation. Additionally, we and others may utilize an IL-13 treatment or allergen challenge model in Tollip deficient mice, in conjunction with the STAT3 inhibitor to determine the in vivo role of Tollip and STAT3 interaction in type 2 inflammation.

## Conclusions

The current study has further demonstrated Tollip as an important regulator in human diseases. Our discovery that Tollip deficiency promotes airway type 2 inflammatory response possibly through the interaction or inhibition with STAT3 likely changes the therapeutic strategies (e.g. use of a STAT3 inhibitor) in treating patients with Tollip deficiency.

## Data Availability

The datasets used and/or analyzed during the current study are available from the corresponding author on reasonable request.

## Abbreviations

Tollip: Toll-interacting protein
Scr. Ctrl.: Scrambled control
KD: Knockdown
HTBE: Human tracheobronchial epithelial
P-STAT3: Phosphorylated STAT3
Co-IP: Co-immunoprecipitation
